# Bilateral engagement of the occipito-temporal cortex in response to dance kinematics in experts

**DOI:** 10.1038/s41598-018-37876-x

**Published:** 2019-01-30

**Authors:** Andrea Orlandi, Alice Mado Proverbio

**Affiliations:** Neuro-MI, Milan Center for Neuroscience, Department of Psychology, University of Milano - Bicocca, Milan, Italy

## Abstract

Previous evidence has shown neuroplastic changes in brain anatomy and connectivity associated with the acquisition of professional visuomotor skills. Reduced hemispherical asymmetry was found in the sensorimotor and visual areas in expert musicians and athletes compared with non-experts. Moreover, increased expertise with faces, body, and objects resulted in an enhanced engagement of the occipito-temporal cortex (OTC) during stimulus observation. The present study aimed at investigating whether intense and extended practice with dance would result in an enhanced symmetric response of OTC at an early stage of action processing. Expert ballet dancers and non-dancer controls were presented with videos depicting ballet steps during EEG recording. The observation of the moving dancer elicited a posterior N2 component, being larger over the left hemisphere in dancers than controls. The source reconstruction (swLORETA) of the negativity showed the engagement of the bilateral inferior and middle temporal regions in experts, while right-lateralized activity was found in controls. The dancers also showed an early P2 and enhanced P300 responses, indicating faster stimulus processing and subsequent recognition. This evidence seemed to suggest expertise-related increased sensitivity of the OTC in encoding body kinematics. Thus, we speculated that long-term whole-body practice would result in enriched and refined action processing.

## Introduction

Research has shown structural and functional changes in the brains of highly skilled athletes and musicians as a result of intensive visuomotor practice^1,2^. Voxel-based morphometry (VBM) and diffusion tensor imaging (DTI) studies have found plastic modulations of brain regions and connections underlying the acquisition of specific cognitive and physical abilities. These processes are also affected by the age of acquisition and expertise level^3,4^. For instance, handball players and ballet dancers show increased grey matter (GM) volume in the primary motor and sensory regions, along with a reduction of fractional anisotropy (FA) in the corticospinal tracts^3^. Specifically, these modulations are evident in the cortical areas related to the representation of the hand in handball players, and of the foot in dancers. Previously, Hüfner and colleagues^4^ showed volume reduction in the anterior parahippocampal formation and parieto-vestibular cortex in experts (dancers and slackliners) compared with non-experts, which is likely associated with the suppression of destabilizing vestibular input. At the same time, increased GM volume in the posterior parahippocampal formation and visual regions (fusiform and lingual gyri) of experts suggests greater use of visual information for balance. More importantly, evidence shows specific changes in the structure of the corpus callosum (CC) in professional musicians^5^ and dancers^6,7^, suggesting increased interhemispheric communication as a result of intensive training. In this regard, Schlaug and colleagues^5^ showed an enlargement of the anterior mid-body of the CC (connecting premotor and supplementary motor areas of the two hemispheres) in young musicians (compared with non-musicians) depending on the hours of music practice (high-practice vs. low-practice). In addition, behavioral evidence supports the hypothesis of an expertise-related reduction of hemispherical asymmetries in visuomotor regions. For instance, musicians with an earlier age of acquisition show greater costs in reaction times during bimanual compared with unimanual tasks^8^. Furthermore, expert fencers exhibit similar accuracy using both hands during a target reaching task, while non-experts show better performance using the dominant (right) hand. Experts also show increased preference for their non-dominant hand when provided with no instruction on which hand to use^9^. Finally, non-dancers exhibit improved accuracy in estimating the position of one hand when both limbs (compared with one limb) are passively moved. Contrarily, dancers show similar performance abilities in both bimanual and unimanual conditions, which is likely the result of increased interhemispheric inhibition capability^10^. Previous evidence has demonstrated improved proprioceptive abilities^11^ and sensorimotor body representation^12,13^ in highly skilled athletes.

Several studies suggest that this greater functional symmetry in musically trained individuals is extended beyond visuomotor regions. In an fMRI study by Burunat and colleagues^14^, music listening engaged more bilateral activity in the brains of musicians than non-musicians. This network includes the sensorimotor regions (paracentral lobule, pre- and postcentral gyri), calcarine fissure, precuneus, inferior/superior temporal and fusiform gyrus, orbitofrontal cortex, cerebellum, and a few subcortical structures (caudate nucleus and putamen). These symmetries are more prominent in keyboard compared with string players, possibly due to an analogous use of the two hands in pianists. Furthermore, a similar reduction in asymmetry has been identified in the visual regions of musicians, which is likely the result of improved score reading skills. Patston and colleagues^15^ showed that lateralized visual targets (checkboard) elicited an earlier posterior N1 component (event-related potential, ERP) over the left than right hemisphere in non-musicians. Moreover, the interhemispheric transfer time (computed as the difference in N1 latency between hemispheres) was faster in the right-to-left than left-to-right direction. Contrarily, no hemispheric difference in latency (or direction) was shown in professionals, which suggests increased connectivity between the two hemispheres. Finally, in a study by Proverbio and colleagues^16^_,_ participants (musicians and controls) were presented with words and short music scores during a target (letters or notes) detection task. Target letters elicited a larger N170 component over the left than right occipito-temporal cortex (OTC) in non-experts, while a bilateral response to both letters and notes was found in experts. The source reconstruction (swLORETA) of this negativity confirmed left-lateralized engagement of the fusiform gyrus (visual word form area) in controls, but bilateral activity in musicians in response to both classes of stimuli. Overall, the evidence presented so far indicates reduced lateralization of cognitive functions and engagement of the related neural substrates, as a function of intense and extended visuomotor practice.

Given the above, in the present investigation, we speculated whether extended dance training could engage a bilateral brain network during action observation, with a specific focus on the early response of the OTC. In this regard, several fMRI studies have shown selective activation (within the OTC) of the extrastriate^17^ (EBA) and fusiform body area^18,19^ (FBA) in response to static and dynamic bodies (when compared with faces, animals, and objects). Similarly, electrophysiological^20–22^ and magnetophysiological^23^ investigations have found that the observation of an intact (than scrambled) body (or body parts) elicits a negative potential (ERP, event-related potential) at approximately 190 ms (N190/M190 component) over occipito-temporal regions of the scalp. This is also confirmed by intracranial recording at a specific electrode site compatible with (right) EBA location^24,25^. More importantly, previous evidence has shown a modulation of OTC response due to increasing visuomotor expertise with observed actions. For instance, a greater recruitment of the EBA was shown when experts (basketball players), compared with non-experts, were asked to predict the outcome of technical gestures^26,27^, or while they observed well-known actions^28,29^. Furthermore, two associative visual regions have shown specific engagement during motion observation. Partially overlapping with the EBA, the hMT + region is linked to motion perception^30,31^, while the pSTS is specifically associated with biological motion processing^32–34^. In this regard, White and colleagues^34^ showed that the observation of upright and inverted point-light animations depicting human actions elicited an N2 component over occipito-temporal sites related to scrambled motion. The N2 was also visible in respose to stationary human forms (i.e., stick figures, upright static point-light figures). Thus, the authors interpreted this negativity as an index of the activity of the EBA/FBA in integrating form and action information, rather than mere biological motion processing, in accordance with Jastorff and Orban^35^. This evidence is also consistent with recent studies using a MVPA (multivoxel pattern analysis) apporach to fMRI^36^ and MEG^37^ data, showing an engagement of the OTC in action encoding.

It is interesting to note that patients suffering from body image disorders (i.e., bulimia and nervous anorexia) show reduced engagement of the left OTC during the observation of images of their own distorted bodies^38^ (i.e., fatter or thinner than the original). A reduction of GM volume of the left EBA^39^, and reduced connectivity between the left FBA and the EBA^40^ has also been identified in this clinical population. Contrarily, increased activity in the left EBA was found after therapy focusing on the restoration of body representation awareness^41^, which possibly suggests more intense body image processing after therapy. Finally, the activity of the OTC (i.e., fusiform gyrus) is also modulated by increased visual expertise^42^ with known^43^ (i.e., cars) or novel objects^44^ (i.e., Greebles). In this regard, Scott and colleagues^45,46^ showed improved accuracy in object categorization after subordinate-level visual training with cars, supported by a larger posterior N2 component (250 ms). Moreover, the modulation of this negativity was persistent a week after training. Thus, this component is interpreted as an index of acquired long-term perceptual expertise.

**Figure 1:**
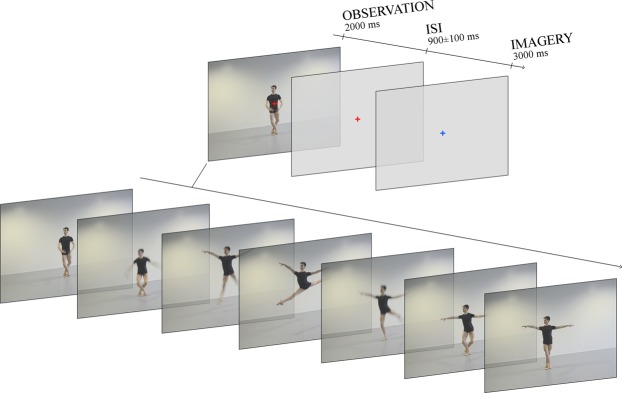
Timescale of the experimental design. Each experimental trial consisted of an observation phase (2000 ms) and subsequent motor imagery phase (3000 ms). During the observation, each video depicting a technical dance step was presented at the center of the screen. It was then followed by an interstimulus interval (ISI) lasting 900 ± 100 ms, in which a red cross on an isoluminant light grey background was shown. The motor imagery phase started as soon as the fixation cross turned into blue. An example of stimuli is visible in the left lower corner of the figure. Seven static frames from a video are presented here overlapping each other to illustrate the typical timing of a movement: preparation (onset), climax (1000 ms), end (2000 ms).

In this study, brain bioelectrical activity (electroencephalography, EEG) of expert ballet dancers and non-dancer controls were compared during action observation to determine whether ballet training results in a modulation of the OTC response to a moving body. Dance expertise in our study included both motor and visual familiarity with the observed gestures. Although a decoupling between these two aspects was not one of the aims of the experiment, previous research has shown the distinct impact of motor knowledge and visual exposure to action repertoire during action observation^47^. Our participants were presented with 326 short videos depicting technical ballet steps and were instructed to carefully observe each clip in order to mentally reproduce the movements (see Fig. 1). Three ERP components were considered and analysed: the previously described N2 response over occipito-temporal regions, the earlier P2 potential, and the subsequent fronto-central P300 component. The P2 and N2 responses have generally been identified over posterior sites of the scalp during visual tasks. They are considered an index of stimulus processing^48–50^ and are modulated by attention allocation and expertise^51^. The P300 is classically associated with stimulus recognition, categorization, and visual awareness^52,53^. We therefore hypothesised finding a larger N2 component over the left hemisphere in dancers compared with controls, as an index of greater bilateral involvement of OTC in moving body processing due to acquired expertise^45,46,54^. Furthermore, an earlier P2 response was expected in dancers together with a larger P300, as a result of faster perceptual coding of dance gestures and consequent recognition^55–57^. Finally, a swLORETA source reconstruction was performed (in the N2 time window) to estimate the neural sources of the negative potential recorded over the scalp. Engagement of the inferior temporal (FG) and extrastriate cortex was expected, being specifically more bilateral in expert dancers^27,28^ than controls^58^.

## Results

### P2

#### Latency

**Figure 2:**
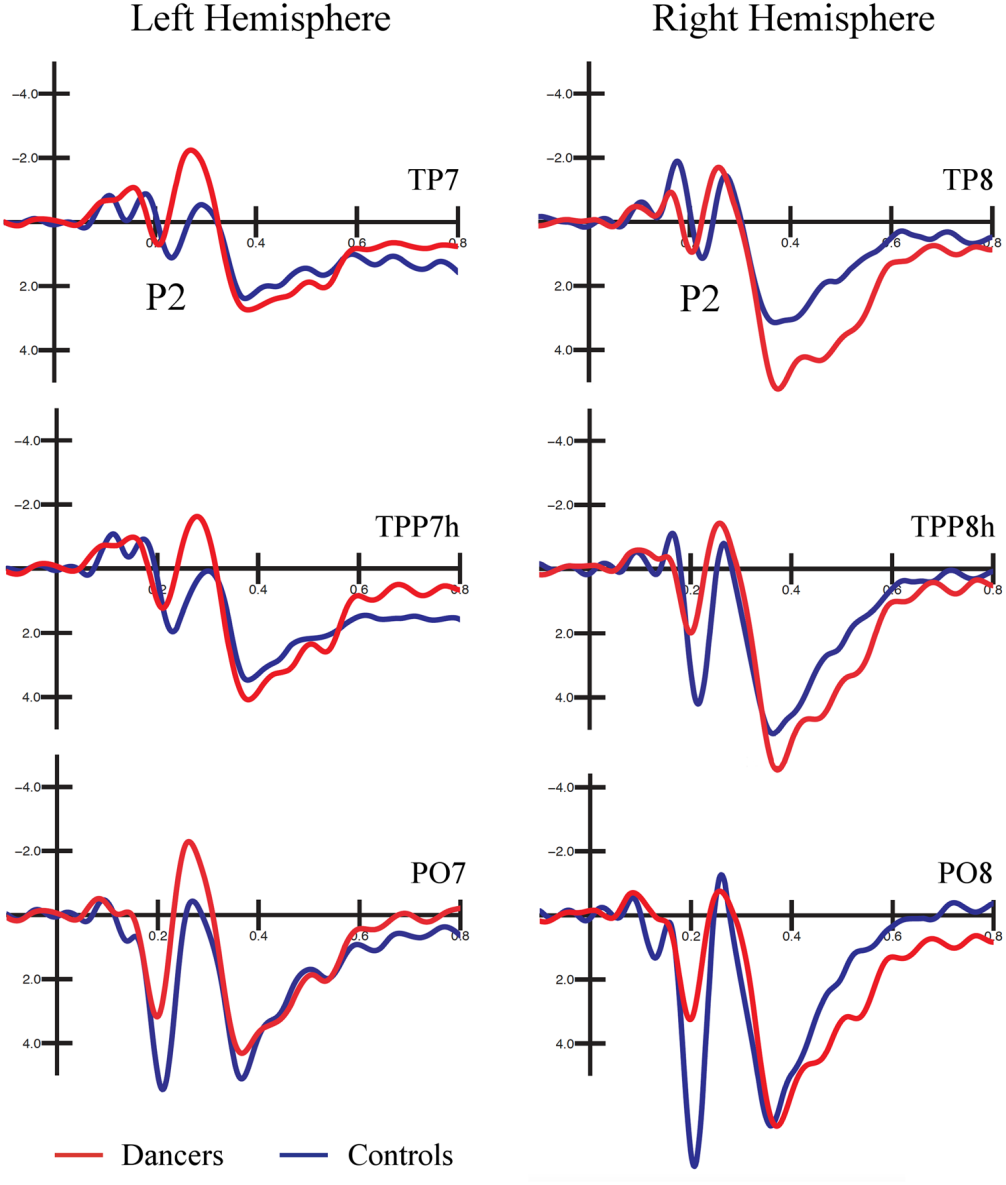
Grand average waveforms recorded at occipito-temporal and temporo-parietal sites. Grand average waveforms (ERPs) recorded over occipito-temporal and temporo-parietal sites in dancers (in red) and controls (blue). An early P2 response is visible in the dancers than controls at all electrode sites. A between-group difference in the P2 amplitude was also found at PO7-PO8 electrode sites, being larger in the controls than dancers.

As can be appreciated in Fig. 2, the latency of the P2 was modulated by the expertise of the participants. The ANOVA performed on the P2 latency values showed a main effect of the group factor [F(1, 32) = 7.903, *p* < 0.01]. The P2 response was faster in the dancers (203 ms, SE = 3.83) than controls (218 ms, SE = 3.83). Furthermore, the significant electrode factor [F(2, 64) = 14.162, *p* < 0.0001] and relative post-hoc tests indicated that the P2 was faster over posterior occipito-temporal sites (PO7-PO8: 204 ms, SE = 2.79) than anterior occipito-temporal sites (TPP7h-TPP8h: 211 ms, SE = 2.94, p < 0.003; TP7-TP8: 216 ms, SE = 3.36, p < 0.0001). Finally, the P2 was faster over the right (206 ms, SE = 2.99) than left (214 ms, SE = 3.09) hemisphere, as shown by the significant hemisphere factor [F(1, 32) = 7.963, *p* < 0.01].

#### Amplitude

**Figure 3:**
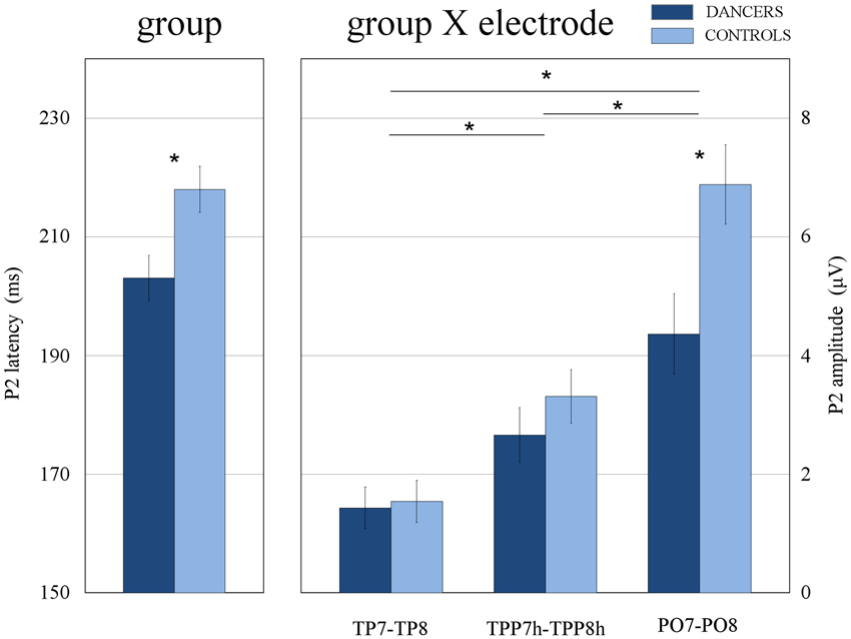
Latency and amplitude values of the P2 component. The histogram on the left displays the latency values (ms) of the P2 component recorded over occipito-temporal and temporo-parietal sites as a function of the group factor. A larger positivity was shown in the brain of the dancers compared with controls. The histogram on the right displays the peak amplitude values (μV) of the P2 component as a function of the group and electrode factors. A larger positivity was found in the control group compared with the dancers’ group only over PO7-PO8 electrode sites.

The ANOVA performed on the P2 amplitude values showed the significance of the electrode factor [F(2, 64) = 66.402, *p* < 0.0001]. The P2 component was larger over posterior occipito-temporal sites (PO7-PO8: 5.62 μV, SE = 0.47) than anterior occipito-temporal sites (TPP7h-TPP8h: 2.99 μV, SE = 0.32; TP7-TP8 (1.49 μV, SE = 0.25). The relative post-hoc tests showed a significant difference between all electrode sites (p < 0.001). The further group X electrode interaction [F(2, 64) = 6.054, *p* < 0.01] and relative post-hoc comparisons confirmed the former difference in both group of participants (see Fig. 3). More importantly, the P2 was larger in controls (6.88 μV, SE = 0.67) than dancers (4.36 μV, SE = 0.67) only over the posterior sites (PO7-PO8: p < 0.001), but not anterior sites (TP7-TP8: p = 0.88; TPP7h-TPP8h: p = 0.37). The significant electrode X hemisphere interaction [F(2, 64) = 3.753, *p* < 0.03] also showed that the P2 was larger over the right than left hemisphere at PO7-PO8 (right: 6.28 μV, SE = 0.71; left: 4.97 μV, SE = 0.55; p < 0.008) and TPP7h-TPP8h (right: 3.79 μV, SE = 0.50; left: 2.19 μV, SE = 0.32; p < 0.002), but not TP7-TP8 (p = 80), as confirmed by the post-hoc tests.

### N2

#### Latency

The ANOVA performed on N2 latency values showed that the N2 response was earlier over the right (263 ms, SE = 3.09) than the left hemisphere (274 ms, SE = 3.71), as shown by the significance of the hemisphere factor [F(1, 32) = 9.800, *p* = 0.0037]. Moreover, the N2 potential reached the maximum peak earlier over posterior occipito-temporal sites (PO9-PO10: 266 ms, SE = 3.21; PPO9h-PPO10h: 267 ms, SE = 3.12) than anterior occipito-temporal sites (P7-P8: 271 ms, SE = 2.82; TPP9h-TPP10h: 271 ms, SE = 3.01), as provided by the statistically significance of electrode factor [F(3, 96) = 6.705, *p* < 0.001] and the relative Duncan post-hot comparisons.

Finally, the further hemisphere X electrode interaction [F(3, 96) = 5.953, *p* < 0.001] and the relative Duncan post-hoc comparisons showed that overall the N2 was earlier over the right than left hemisphere at all electrode sites (*p* < 0.001). The difference in latency between electrode sites was visible only over the left hemisphere, with a later N2 response at more posterior than anterior occipito-temporal sites (*p* < 0.001). Contrarily, no difference between electrode sites was found over the right hemisphere (*p* > 0.50).

#### Amplitude

**Figure 4:**
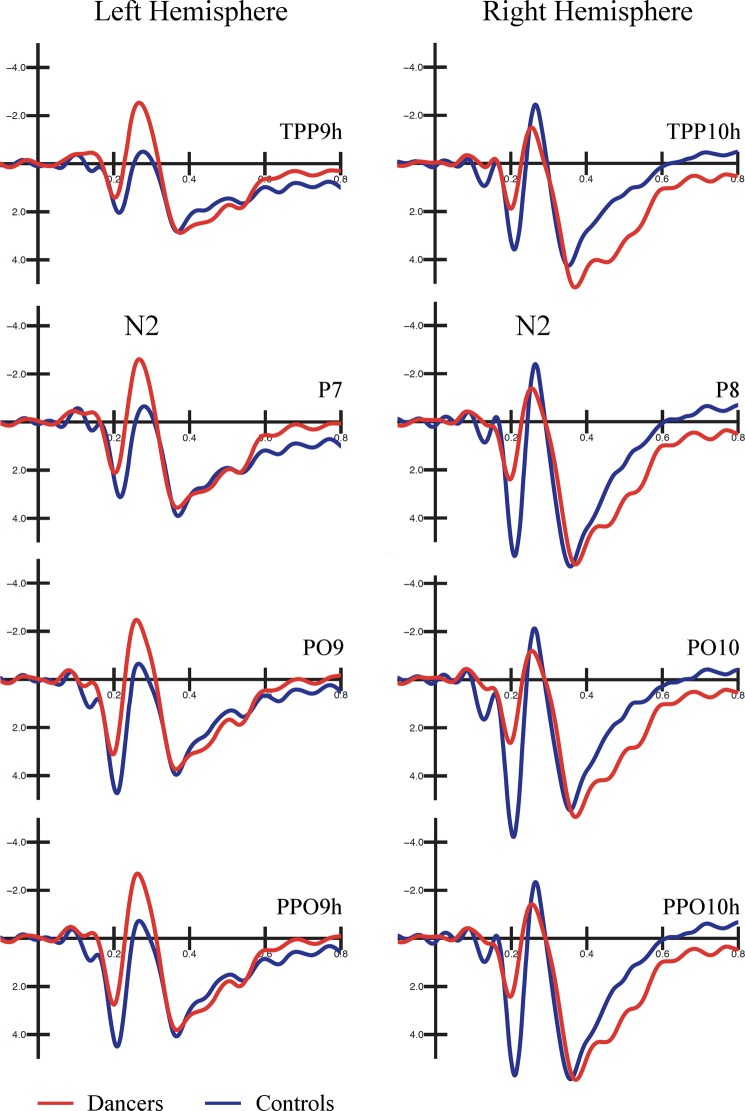
Grand average waveforms recorded at occipito-temporal sites. Grand average waveforms (ERPs) recorded over occipito-temporal sites in dancers (in red) and controls (in blue). An expertise-related modulation of the N2 component is visible over the left hemisphere, being larger in the dancers than controls.

**Figure 5:**
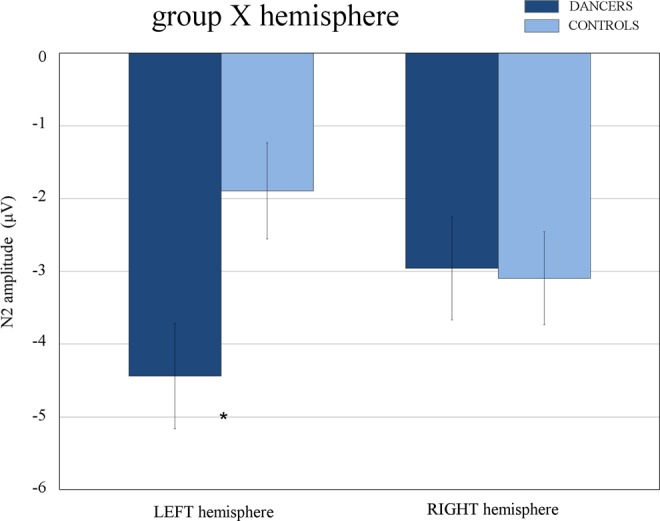
Amplitude values of the N2 component. Peak amplitude values (μV) of the N2 component recorded at occipito-temporal sites as a function of the hemisphere and group factors. A larger negativity (N2) was found over the left hemisphere in the brain of the dancers compared with controls. Contrarily, no expertise-related difference was shown over the right hemisphere.

**Figure 6:**
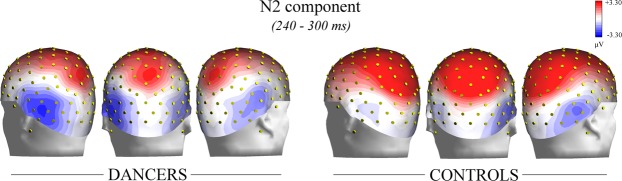
Topographic maps of voltage distribution over the scalp. Topographic maps of scalp voltage distribution of the N2 response (240–300 ms) recorded in dancers (on the left) and controls (on the right). The negative values of voltage are shown in blue, while the positive values in red. The negative potential (N2) is visible bilaterally over the occipito-temporal regions in the brains of dancers, while it is more localized over the right hemisphere in controls.

As can be seen in Figs 4 and 5, a different modulation of ERP waveforms based on acquired expertise with ballet was shown over occipito-temporal sites. The ANOVA performed on N2 amplitude values, and the relative Duncan post-hoc test showed the significance of the group X hemisphere interaction [F(1, 32) = 5.269, *p* < 0.03]. Namely, the negativity was larger in dancers’ (−4.44 μV, SE = 0.72) than controls’ (−1.89 μV, SE = 0.70) group (*p* < 0.04) over the left hemisphere. Contrarily, no difference between groups was found over the right hemisphere (dancers: −2.96 μV, SE = 0.66; controls: −3.09 μV, SE = 0.64*; p* = 0.90). This result is also visible in Fig. 6, which illustrates the topographical maps (back view) of the current distribution over the scalp (in the N2 time window: 240–300 ms) for experts (on the left) and non-experts (on the right). The maximum peak of negativity (represented in blue) is visible over both hemisphere of dancers, while more right lateralization can be appreciate in controls.

### swLORETA source reconstruction (240–300 ms)

**Figure 7:**
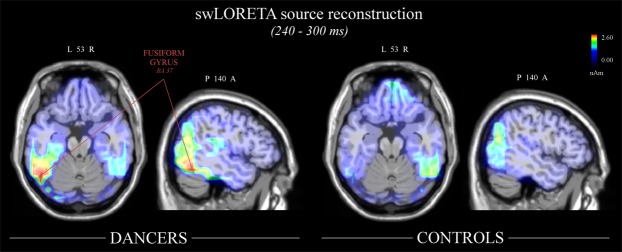
SwLORETA source reconstruction of surface potentials in the N2 time window (240–300 ms). SwLORETA performed on the grand-average waveforms (in the N2 time window 240–300 ms) in dancers (left side of the figure) and controls (right side of the figure). The axial and sagittal anatomical planes of the brain are shown. The activation of the left fusiform gyrus (BA 37) is visible only in the brains of dancers. The strongest magnitude values of the signal (nAm) are presented in red.

Two swLORETA source reconstructions were performed to solve the so-called inverse problem and estimate the neural sources of the surface potential recorded over the scalp in the two groups of participants. The time window between 240 and 300 ms was selected to include the peaks of N2 component over the left and right hemispheres in dancers and controls. A list of significant active electromagnetic dipoles for both groups of participants is shown in Table 1. The main active dipoles included bilateral body- and face-related visual regions, such as the fusiform gyrus (BA 37/20) and middle temporal gyrus (39/21), cuneus/precuneus (BA 19) in expert dancers. In addition, activity in the right fronto-parietal mirror regions (inferior parietal lobule, BA 40 and precentral gyrus, BA 6) and superior and medial frontal gyri (BA 9/10) was found in the brains of dancers. Conversely, the right hemisphere was mainly engaged in the brains of controls, including the cuneus (BA 19), superior and medial frontal areas (BA 10), precentral (BA 6) and left postcentral gyri (BA 3). Figure 7 shows the estimated active dipoles in the left fusiform regions found in the brains (presented in horizontal and sagittal axis) of the dancers compared with controls. The maximum values of magnitude (nAm) of the sources are represented in red.

**Table 1:** List of active electromagnetic dipoles.

Dancers								
*Magnitude*	*T-x [mm]*	*T-y [mm]*	*T-z [mm]*	*Hem*	*Lobe*	*Gyrus*	*BA*	*Function*
26.69	−48.5	−55.0	−17.6	L	T	Fusiform Gyrus	37	Body and face related visual regions
25.99	−38.5	−69.0	13.6	L	T	Middle Temporal Gyrus	39	
22.94	−8.5	−81.1	30.6	L	O	Cuneus	19	
22.21	11.3	−82.1	39.5	R	P	Precuneus	19	
18.16	50.8	−33.7	−23.6	R	T	Fusiform Gyrus	20	
17.76	60.6	−35.7	−8.8	R	T	Middle Temporal Gyrus	21	
18.01	60.6	−39.6	25.1	R	P	Inferior Parietal Lobule	40	Sensorimotor regions
9.47	40.9	2.4	29.4	R	F	Precentral Gyrus	6	
15.97	1.5	53.4	24.8	R	F	Superior Frontal Gyrus	9	
15.03	21.2	53.4	24.8	R	F	Superior Frontal Gyrus	9	
13.07	1.5	57.3	−9.0	R	F	Medial Frontal Gyrus	10	Working memory
**Controls**								
***Magnitude***	***T-x [mm]***	***T-y [mm]***	***T-z [mm]***	***Hem***	***Lobe***	***Gyrus***	***BA***	***Function***
36.25	11.3	−81.1	30.6	R	O	Cuneus	19	Body and face related visual regions
21.09	11.3	44.4	15.0	R	F	Medial Frontal Gyrus	10	Working memory
20.58	31.0	54.4	15.9	R	F	Superior Frontal Gyrus	10	
18.75	1.5	57.3	−9.0	R	F	Medial Frontal Gyrus	10	
14.68	40.9	2.4	29.4	R	F	Precentral Gyrus	6	Sensorimotor regions
9.08	−38.5	−21.0	35.7	L	P	Postcentral Gyrus	3	

List of active electromagnetic dipoles identified in dancers and controls according to swLORETA in the N2 time windows (240–300 ms), with the relative Talairach coordinates. The strongest sources of activation included the fusiform and middle temporal gyri bilaterally, cuneus/precuneus and right inferior parietal lobule in dancers. The main dipoles in controls were instead localized in the right cuneus and anterior prefrontal cortex. (Legend: Hem - hemisphere, T - temporal lobe, P - parietal lobe, F - frontal lobe, O - occipital lobe, BA - Brodmann area).

### P300 (360–560 ms)

**Figure 8:**
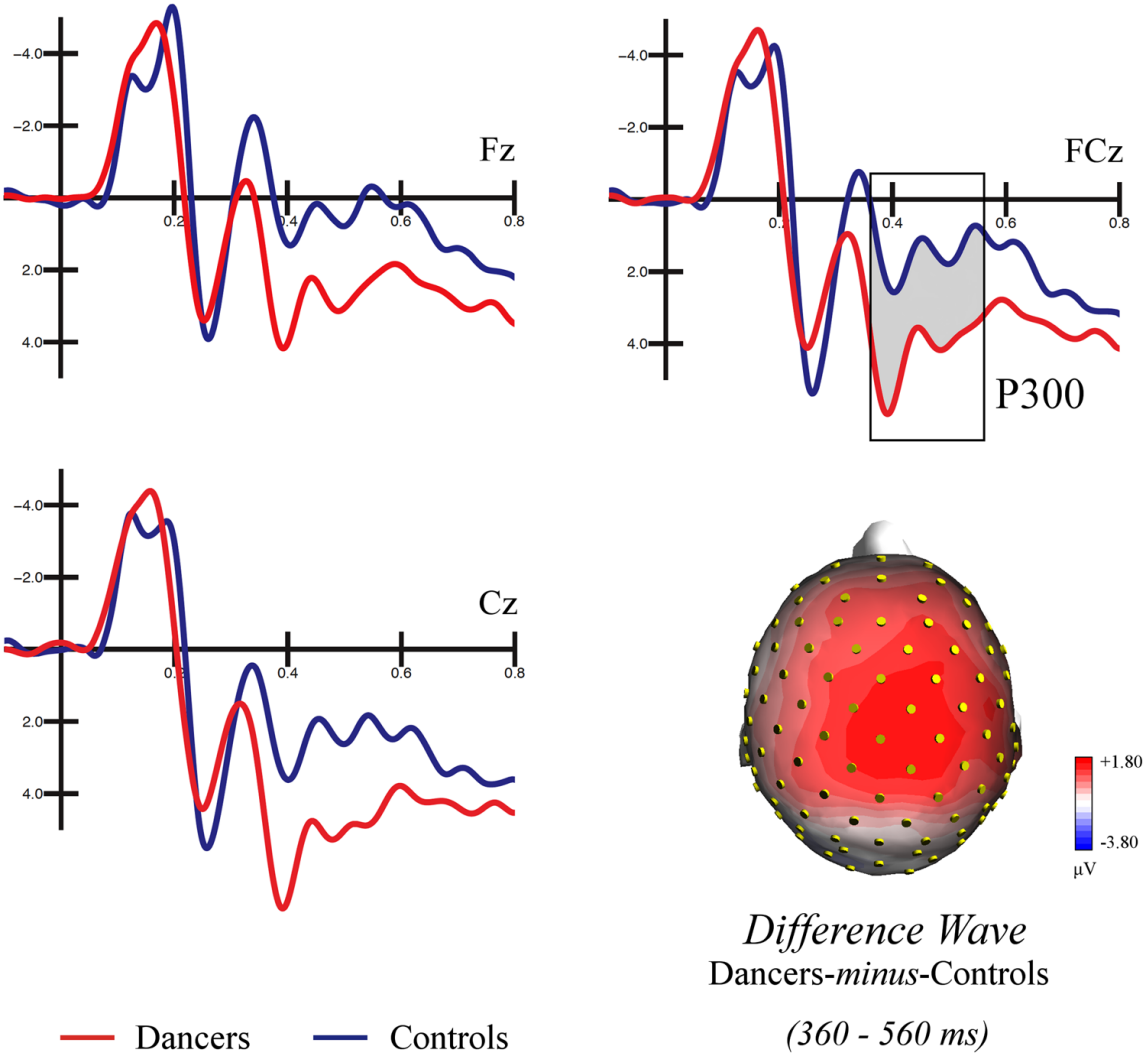
Grand average waveforms recorded at fronto-central sites and topographic map of voltage distribution over the scalp. Grand average waveforms (ERPs) recorded over fronto-central sites in dancers (in red) and controls (in blue). An expertise-related modulation of the P300 component is visible over the midline, being larger in the dancers than controls. The topographic map of the scalp voltage distribution computed on the difference wave dancers-*minus*-controls in the P300 time window (360–560 ms) is shown in the bottom-right part of the figure. The positive values of voltage are shown in shades of red.

As can be appreciated in Fig. 8, the fronto-central P300 was modulated by dance expertise. The ANOVA performed on the mean area of the P300 indicated a larger positivity in dancers (3.27 μV, SE = 0.56) than controls (0.96 μV, SE = 0.56), as shown by the significant group factor [F(1, 32) = 8.538, *p* < 0.007]. Moreover, the significant electrode factor [F(2, 64) = 53.058, *p* < 0.0001] and relative post-hoc tests showed that the P300 was larger over central than frontal sites (Cz: 2,99 μV, SE = 0.41; FCz; 2.12 μV, SE = 0.41; Fz: 1.23 μV, SE = 0.41; p < 0.001).

## Discussion

The present electrophysiological study investigated the time course and neural correlates of the OTC response to dance gesture perception, as a function of acquired dance skills. During EEG recording, professional female dancers and non-dancer controls were shown videos of movements from ballet repertoire. Participants were instructed to observe each video clip and to subsequently imagine reproducing the movements. Evidence shows distinct regions in the OTC selectively activate during body, face, and object perception^59–61^, with a larger engagement of the EBA and the FBA in the right than left hemisphere^17^. Additionally, changes in body awareness (e.g., patients with eating disorders) or increased visual expertise with a category of objects^30,34^, lead to a modulation in the activity of the left OTC. Moreover, motion (hMT+)^31^ and biological motion (pSTS)^32,33^ sensitive regions have been found in the OTC, together with a specific engagement of these areas in action encoding^36,37^.

In the present study, as can be appreciated in the ERP waveforms (see Fig. 4) and maps of the topographic distribution of the voltage on the scalp (see Fig. 6), the amplitude of N2 over the left OTC differed based on ballet expertise level. In particular, the amplitude was larger in expert dancers compared with controls over the left hemisphere, while no difference between groups was visible over the right hemisphere (see Fig. 5). This negative potential is generally found between 230 and 300 ms over posterior sites of the scalp and is considered an index of attentional modulation of the visual cortex^49,62,63^. It can be elicited during visual search tasks and rare target detection paradigms. For instance, a larger N2 component was shown in response to attended (target) rather than unattended features of stimuli, including shape, color, size and face gender^48,64,65^.

The difference wave obtained by subtracting ERPs to non-target from those elicited by the target is usually referred to as selection negativity (SN), and is used as a measure of selective attention allocation processes. In our investigation, all the stimuli were targets as participants had to focus on the body and kinematics presented in each video, encode as many details as they could, and then mentally simulate the same movement. As previously anticipated, a modulation of the N2 component was found as a result of increased expertise with specific visual stimuli. For instance, Scott and colleagues^46^ trained volunteers with images of cars at basic (category of cars) or subordinate (specific items) level. During a same-different judging task, a larger N170 was shown in response to cars trained with both modalities. Contrarily, only the N2 was enhanced for subordinate-level trained stimuli, which also persisted in a re-test after a week, according to previous evidence obtained with pictures of animals^45^. In an fMRI study by Wong and colleagues^66^, a similar dissociation between training modalities was found, showing an increased response in the right FG for subordinate-level individuation and in medial (than lateral) ventral OTC for basic-categorization of novel abstract objects. This suggests an increased response to trained stimuli with increasing detail encoded.

The modulation of the N2 potential was also shown in response to face familiarity^54^. Repeated exposure to a specific face led to a larger N250 over the left hemisphere compared with non-trained faces, similarly to the negativity evoked by the observation of the participant’s face. Recently, Folstein and colleagues^67^ proposed an additive effect of expertise-related N250 over attention-related SN, suggesting an overlap between two different cognitive processes. More importantly, a posterior negative response was found in several investigations of biological motion perception^30,32,33^. The latency of this component differed between these aforementioned studies^30,32,33^ (i.e., 240 ms, 330 ms) but is compatible with the time window of our N2. This negativity was enhanced in response to point-light animations depicting biological motion rather than scrambled motion. Motion-selective attention also enhanced this response, that was bilaterally distributed but with a predominance over the right hemisphere^33^.

White and colleagues^34^ showed a modulation of the N2 also in response to static human figures, thus interpreting the negativity as an index of shape and action integration. This is consistent with research by Jastorff and Orban^35^, that showed the automatic engagement of the EBA and FBA, even in the absence of a task, in response to human shape and action kinematics. The authors suggested that these two regions of the OTC are involved in the initial stage of action processing, linking the action to the body of the actor. Thus, we speculated that the modulation of our N2 was possibly related to acquired expertise with moving bodies, including both motor knowledge and visual familiarity with the ballet steps. After years of performances, dancers likely develop a refined ability to process body kinematics in order to rapidly learn new structures of complex whole-body movements. In this regard, the classical literature on action observation suggests a main contribution of the OTC regions in low-level feature processing^68^, with a subsequent integration of this information within a wider cortical network (i.e., action observation network) engaged in action understanding^69,70^.

However, other authors emphasize the role of the OTC in action representation^37,71^ and preparation^72^. For instance, using multivariate pattern analysis applied to MEG data, Tucciarelli and colleagues^37^ showed early decoding of action intention (reaching vs. grasping) through the response of lateral OTC (200–600 ms), followed by later decoding in the precentral regions (600–1200 ms). Moreover, increased activity in the posterior and ventral OTC was found during the observation of typical (than atypical) object-directed grasping actions^73^. An enhanced response of the middle temporal gyrus (in addition to premotor and anterior ventrolateral prefrontal cortices) was also found during the judgment of the goals of point light animations^74^, but only for a simple task condition (low noise). Prefrontal regions were instead engaged during increasing task difficulty (from low to high noise). This seems to suggest a confluence of action-related visual, motor, and semantic information within occipito-temporal brain regions^75^.

In the present study, to further estimate the neural generators of the N2 potential, a swLORETA source reconstruction was performed separately on the grand-average waveforms in dancers and controls, between 240 and 300 ms (N2 time window). The main active electromagnetic dipoles (see Table 1) were bilaterally localized in the fusiform gyrus (BA 37/20), middle temporal gyrus (BA 39/21), and precuneus/cuneus (BA 19) in dancers, while the right cuneus (BA 19) was identified in non-dancers. These results are consistent with previous evidence showing a specific response of the EBA and the FBA within the middle and inferior temporal gyri to the human body^18,19,76^. Moreover, our N2 potential was recorded over electrode sites generally used for N190, whose neural sources were localized in the EBA^21,23^.

Furthermore, the engagement of a larger number of right regions in expert dancers (than controls) was consistent with enhanced activity in the right OTC found in response to familiar animals and objects^43,66^. On the other hand, the recruitment of regions in the left hemisphere of dancers (see Fig. 7) could be the result of increased visuomotor expertise and body awareness^12,41^. Indeed, reduced activity in the left EBA was found in clinical patients suffering from body image disorders, along with reduced functional connectivity between the EBA and the FBA^39,40^. This suggests a specific sensitivity of the left OTC to body awareness. In addition, several visuomotor regions were engaged in the right hemisphere in our experts, including the inferior parietal lobule (BA 40), precentral (BA 6) and superior frontal (BA 9) gyri (SFG). The contribution of fronto-parietal visuomotor regions to action representation is well-established in the literature and has strengthened the idea of visuomotor coding of dance kinematics in experts. IPL^29^, vPM^28,77^ and SFG^78^ are generally engaged during action observation, with a greater activity in response to trained (than untrained) and well-known movement repertoire. TMS studies have also shown that stimulation of vPM leads to reduced accuracy during a matching-to-sample task with upright (but not inverted) body postures, suggesting a role of vPM in configurational body processing^79^. At the same time, disruption of vPM results in an increased contribution of the EBA in local body processing, thus increasing aesthetic sensibility^80^. In the present study, the right anterior prefrontal cortex (BA 10) and the precentral (BA 6) and postcentral gyri (BA 3), were instead active in controls. Engagement of these prefrontal regions was previously found when a coordination of multiple cognitive operations was required^81–83^, demonstrating working and prospective memory processes^84,85^. In our study, this possibly suggests a high cognitive load required by controls to perceive and integrate body kinematics into a complex movement. In a MEG study by Kietzmann and colleagues^86^, the extensive visual training with new categories of objects (artificial faces) resulted in a spatiotemporal shift of the activity within cortical regions during object categorization, from prefrontal to occipito-temporal areas. This was likely the result of a more efficient (quick and reliable) recognition processing for recurrent stimuli.

Expertise-related differences were also found in the posterior P2 response, as can be seen in Figs 2 and 3. The P2 component has been linked to perceptual analysis of the stimulus, as shown by previous studies on face, object, and implied motion perception^50,87,88^. The P2 latency has been interpreted as an index of the time required for perceptual processing, with less efficient coding characterized by slower peaks^55,56^. Here we found an earlier P2 latency in dancers (203 ms) compared with controls (218 ms), suggesting a faster and more effective encoding of dance kinematics. Contrarily, the controls not only showed slower stimulus processing (later P2), but also, required increased cortical resources as indexed by the P2 amplitude modulation. The component was more positive over the posterior occipito-temporal sites (but not temporo-parietal sites) in non-dancers than dancers. This suggests an enhanced activity of the body-related visual regions in non-expert (compared with expert) observers. Increased P2 amplitude was previously reported during the observation of stimuli (i.e., faces) subsequently better remembered^89^, images depicting great motion content^50^, and during divided-attention tasks^90^. At the same time, a reduction of the P2 was found as a function of increasing expertise^51^. Furthermore, in our study both the P2 and N2 components were faster over the right than left hemisphere, with the P2 also being larger over the right OTC. These results are consistent with previous research showing that, despite a bilateral engagement of OTC in body and motion perception^33,58,91^, the right hemisphere may have a prominent role^17,28^. For instance, Aleong and Paus^58^ showed that the observation of a body varying in size resulted in greater activity in the right OTC in female participants, while a bilateral response was found in male participants.

Finally, the fronto-central distributed P300 recorded over the midline sites was sensitive to dance expertise, since it was larger in dancers compared with controls (see Fig. 8). This positivity is classically interpreted as indexing updating the mental representation of stimulus context, item recognition and categorization^52,53^. EEG studies have shown a modulation of the component as a function of acquired expertise in high-skilled badminton players^57^, dancers^92^, and musicians^93^. For instance, Jin and colleagues^57^ showed enhanced P300 over parietal sites in professional badminton players (compared with non-player controls) when asked to predict the ball’s landing position during game action observation. Thus, we considered the enhanced P300 an indicator of refined gesture recognition in expert observers. At the same time, the reduced amplitude in controls suggests a difficulty in action identification, which is likely the result of a lack of ballet expertise. This result is consistent with previous evidence showing enhanced ability to detect^29^ and categorize action variations^80^ and violations^94^ in professionals than control participants.

In summary, a larger N2 response elicited during the perception of dance steps was found over the left hemisphere in expert dancers compared with non-dancer controls. The source reconstruction of this negativity showed bilateral engagement of the inferior and middle temporal regions in dancers, along with right visuomotor areas. Contrarily, only the right visual and prefrontal regions were active in controls. Furthermore, the dancers (relative to controls) showed an early P2 component followed by a larger P300, suggesting faster gesture processing and enhanced gesture recognition. At the same time, the larger P2 over body-related regions in controls (compared with dancers) indicates greater stimulus encoding, but weaker recognition ability (smaller P300). This suggests that acquired expertise with dance might result in an increased sensitivity of the OTC to dance kinematics, due to extended whole-body practice. The dancers showed more sophisticated and enriched action processing within the first 300 ms once a moving performer was presented compared with non-dancer controls.

## Materials and Methods

### Participants

Thirty-four right-handed female volunteers took part in the present investigation. According to their acquired expertise with ballet, two groups were created (expert dancers vs. non-dancer controls). The group of dancers was composed of 17 professional ballet dancers aged between 20 and 42 (mean age: 25.59 years, SD = 5.29) and with an average of 9 years formal dance training (mean: 8.88 years, SD = 2.80). The mean age of acquisition (the year when they started studying dance) was 5 years (SD = 1.92), stating 20 years (SD = 5.69) of comprehensive acquired ballet expertise. The control group was composed of 17 university students aged between 22 and 30 (mean age: 24.88 years, SD = 2.78) with no experience whatsoever with dance, gymnastics, or martial arts. All the participants had normal or corrected-to-normal vision and no history of drug abuse or neurological illness. Their right-handedness was assessed using the Italian version of the Edinburgh Handedness Inventory (dancers = 0.74; controls = 0.83). In this questionnaire, values between 0.50 and 1 suggest a dominant use of the right hand and foot (but also eye and ear) in executing common human actions (i.e., writing, drawing, kicking a ball). The informed consent of each participant was obtained before the beginning of the experiment. The study received the approval of the ethical committee of the University of Milano-Bicocca and all methods were performed in accordance with the relevant guidelines and regulations.

### Stimuli

The set of stimuli was composed of 326 color videos, each representing one out of six professional male dancers performing a ballet steps. A large variety of technical movements was recorded, all belonging to ballet technique common to both male and female dancers, including turns, jumps, and steps. All movements were initially entirely recorded (using a Nikon D7000 Reflex with a frame rate of 25 fps), from preparation to end (e.g., in the case of a pirouette, from preparation with feet in fifth position to end with feet in fourth position) to ensure precision and accuracy in their execution. The same movement was recorded several times allowing the selection of the best version at a later stage. The selected clips were then silenced (post-production was realized using Adobe Premiere Pro CC 2015, version 9.0) and cut to display 1000 ms before and after the maximum apex of each step (e.g., highest height or larger extension of arms and legs during a jump), resulting in videos lasting 2000 ms. Frames taken from one of the video stimuli can be seen in Fig. 1. The dancer performed at the center of the scene (the camera could be moved on the horizontal axis to follow the dancer), and all body parts were visible. All the dancers wore adherent dark clothes to maximize the visibility of the muscles, reduce body-related differences in low-level features between dancers, and ensure a good contrast with the light grey floor and background of the (empty) rehearsal room. The lighting condition was constant during all the days of recording, ensuring the equiluminance of the stimuli (≅1.75 fL). The definitive videos had a size of 32 × 23 cm, subtending a visual angle of 15°18′ × 11°15′.

### Task and Procedure

Volunteers were seated in an electrically and acoustically shielded cabin, 114 cm away from a high-resolution VGA computer screen. They were instructed to look at fixation cross at the center of the monitor during the experimental sequences, to ensure minimal eyes and body movements. Eevoke v2.2 software (ANT neuro, Enschede, The Netherlands) was used for the stimuli presentation. Each trial included the presentation of a video for 2000 ms, a red fixation cross on an isoluminant light grey background for 900 ± 100 ms (interstimulus interval, ISI), and a blue fixation cross on the same background for 3000 ms. The time scale for an experimental trial is visible in Fig. 1. The participants were instructed to observe the video and mentally simulate the same movement once the change in cross color occurred. The task ensured the maximal attention towards the stimuli, also allowed the investigation of the neural activity underlying motor imagery, as a function of expertise and muscular effort required by the gestures (Orlandi *et al*., *in preparation*). Each stimulus was presented just once during the entire experiment in one out of twelve sequences, which lasted 2.88 minutes, so that the identity of the dancer was different at any trial. The order of the sequences was counterbalanced between participants. Two additional sequences were also created (using extra videos not displayed during the experiment) and used in a training phase to let the volunteers familiarize with both the setting and experimental task (observation and subsequent mental imagery of movements).

### EEG recording and data analysis

EEG was continuously recorded from 128 scalp sites located according to the 10–5 International System^95^ at a sampling rate of 512 Hz, using EEProbe v2.2 software (ANT neuro, Enschede, The Netherlands). Horizontal and vertical eye movements were also recorded. Averaged mastoid (2 electrodes placed over the mastoid bones) served as the reference lead. The EEG and electrooculogram were amplified and filtered with a half-amplitude band-pass of 0.016–70 Hz and a notch of 50 Hz. Electrode impedance was kept below 5 kΩ. Computerized artifact rejection was performed before averaging to discard epochs in which eye movements, blinks, excessive muscle potentials, or amplifier blocking occurred. The artifact rejection criterion was based on peak-to-peak amplitudes exceeding 50 μV. EEGs were also manually inspected to avoid any kind of undetected artifact. EEG epochs were synchronized with the onset of the video. ERPs were averaged off-line from −100 ms before to 2000 ms after stimulus onset and filtered with a band pass of 0.016–15 Hz. ERP components were identified and measured with reference to the average baseline voltage calculated over the 100 ms before the stimulus onset at sites and latency when maximum amplitude was reached^96^, and based on previous literature. ERP averages were computed as a function of the group. The latency and amplitude of the maximum peak of the voltage of the posterior P2 component was measured at TP7, TP8, TPP7h, TTP8h, PO7, and PO8 electrode sites. The latency and amplitude of the maximum peak of the voltage of the occipito-temporal N2 component was measured at TPP9h, TPP10h, P7, P8, PO9, PO10, PPO9H and PPO10h electrode sites. The mean area voltage of the fronto-central P300 component was measured at Fz, FCz, and Cz electrode sites during the 360–560 ms time window. The ERP data were subjected to multifactorial repeated measures ANOVA (performed using Statistica version 10 software by Statsoft) with one between-groups factor (group: dancers, controls) and two within-groups factors, including: hemisphere (left, right) and electrode factors (depending on the component).

Low-Resolution Electromagnetic Tomography (LORETA) was applied to the ERPs elicited during stimuli observation between 240–300 ms in both groups of participants. LORETA, which is a discrete linear solution to the inverse EEG problem, corresponds to the 3-D distribution of neuronal electric activity that yields maximum similarity (i.e., maximum synchronization), regarding orientation and strength, between neighboring neuronal populations (represented by adjacent voxels). In this study, an improved version of standardized weighted low-resolution brain electromagnetic tomography (sLORETA) was used. This version incorporates a singular value decomposition-based lead field weighting: swLORETA^97^. Source space properties included the following: grid spacing (the distance between two calculation points) = 5 points and the estimated signal-to-noise ratio (SNR, which defines the regularization; a higher value for SNR means less regularization and less blurred results) was 3. LORETA was performed on group data to identify statistically significant electromagnetic dipoles (p < 0.05), in which as the magnitude increases, the significance of the group differences increases.

## Data Availability

Anonymized data and details about pre-processing/analyses will be made available to colleagues if requested.

## References

[1] Chang Y (2014). Reorganization and plastic changes of the human brain associated with skill learning and expertise. Frontiers in human neuroscience.

[2] Jäncke L (2009). Music drives brain plasticity. F1000 biology reports.

[3] Meier, J., Topka, M. S. & Hänggi, J. Differences in cortical representation and structural connectivity of hands and feet between professional handball players and ballet dancers. *Neural plasticity*, 2016 (2016). 10.1155/2016/6817397 PMC4876236 27247805

[4] Hufner K (2011). Structural and functional plasticity of the hippocampal formation in professional dancers and slackliners. Hippocampus.

[5] Schlaug G (2009). Training‐induced neuroplasticity in young children. Annals of the New York Academy of Sciences.

[6] Giacosa C, Karpati FJ, Foster NE, Penhune VB, Hyde KL (2016). Dance and music training have different effects on white matter diffusivity in sensorimotor pathways. Neuroimage.

[7] Hänggi J, Koeneke S, Bezzola L, Jäncke L (2010). Structural neuroplasticity in the sensorimotor network of professional female ballet dancers. Human brain mapping.

[8] Hughes CM, Franz EA (2007). Experience-dependent effects in unimanual and bimanual reaction time tasks in musicians. Journal of Motor Behavior.

[9] Akpinar S, Sainburg RL, Kirazci S, Przybyla A (2015). Motor asymmetry in elite fencers. Journal of motor behavior.

[10] Kuehn E, De Havas J, Silkoset E, Gomi H, Haggard P (2015). On the bimanual integration of proprioceptive information. Experimental brain research.

[11] Gautier G, Thouvarecq R, Vuillerme N (2008). Postural control and perceptive configuration: influence of expertise in gymnastics. Gait & Posture.

[12] Jola C, Davis A, Haggard P (2011). Proprioceptive integration and body representation: insights into dancers’ expertise. Experimental Brain Research.

[13] Fourkas AD, Bonavolontà V, Avenanti A, Aglioti SM (2008). Kinesthetic imagery and tool-specific modulation of corticospinal representations in expert tennis players. Cerebral Cortex.

[14] Burunat I (2015). Action in perception: prominent visuo-motor functional symmetry in musicians during music listening. PloS one.

[15] Patston LL, Kirk IJ, Rolfe MHS, Corballis MC, Tippett LJ (2007). The unusual symmetry of musicians: Musicians have equilateral interhemispheric transfer for visual information. Neuropsychologia.

[16] Proverbio AM, Manfredi M, Zani A, Adorni R (2013). Musical expertise affects neural bases of letter recognition. Neuropsychologia.

[17] Downing PE, Jiang Y, Shuman M, Kanwisher N (2001). A cortical area selective for visual processing of the human body. Science.

[18] Peelen MV, Downing PE (2005). Selectivity for the human body in the fusiform gyrus. Journal of neurophysiology.

[19] Schwarzlose RF, Baker CI, Kanwisher N (2005). Separate face and body selectivity on the fusiform gyrus. Journal of Neuroscience.

[20] Taylor JC, Wiggett AJ, Downing PE (2007). Functional MRI analysis of body and body part representations in the extrastriate and fusiform body areas. Journal of neurophysiology.

[21] Taylor JC, Roberts MV, Downing PE, Thierry G (2010). Functional characterisation of the extrastriate body area based on the N1 ERP component. Brain and cognition.

[22] Thierry G (2006). An event-related potential component sensitive to images of the human body. Neuroimage.

[23] Ishizu T, Amemiya K, Yumoto M, Kojima S (2010). Magnetoencephalographic study of the neural responses in body perception. Neuroscience letters.

[24] Engell AD, McCarthy G (2014). Face, eye, and body selective responses in fusiform gyrus and adjacent cortex: an intracranial EEG study. Frontiers in human neuroscience.

[25] Pourtois G, Peelen MV, Spinelli L, Seeck M, Vuilleumier P (2007). Direct intracranial recording of body-selective responses in human extrastriate visual cortex. Neuropsychologia.

[26] Abreu AM (2012). Action anticipation beyond the action observation network: a functional magnetic resonance imaging study in expert basketball players. Eur. J. Neurosci..

[27] Proverbio AM, Crotti N, Manfredi M, Adorni R, Zani A (2012). Who needs a referee? How incorrect basketball actions are automatically detected by basketball players’brain. Scientific reports.

[28] Cross ES, Hamilton AF, de C, Grafton ST (2006). Building a motor simulation de novo: observation of dance by dancers. NeuroImage.

[29] Orlandi A, Zani A, Proverbio AM (2017). Dance expertise modulates visual sensitivity to complex biological movements. Neuropsychologia.

[30] Krakowski AI (2011). The neurophysiology of human biological motion processing: a high-density electrical mapping study. NeuroImage.

[31] Ricciardi E (2007). The effect of visual experience on the development of functional architecture in hMT+. Cerebral Cortex.

[32] Hirai M, Fukushima H, Hiraki K (2003). An event-related potentials study of biological motion perception in humans. Neuroscience letters.

[33] Hirai M, Senju A, Fukushima H, Hiraki K (2005). Active processing of biological motion perception: an ERP study. Cognitive Brain Research.

[34] White NC, Fawcett JM, Newman AJ (2014). Electrophysiological markers of biological motion and human form recognition. Neuroimage.

[35] Jastorff J, Orban GA (2009). Human functional magnetic resonance imaging reveals separation and integration of shape and motion cues in biological motion processing. Journal of Neuroscience.

[36] Ma F (2018). Investigating the neural basis of basic human movement perception using multi-voxel pattern analysis. Experimental brain research.

[37] Tucciarelli R, Turella L, Oosterhof NN, Weisz N, Lingnau A (2015). MEG multivariate analysis reveals early abstract action representations in the lateral occipitotemporal cortex. Journal of Neuroscience.

[38] Mohr HM (2011). Body image distortions in bulimia nervosa: investigating body size overestimation and body size satisfaction by fMRI. Neuroimage.

[39] Suchan B (2010). Reduction of gray matter density in the extrastriate body area in women with anorexia nervosa. Behavioural brain research.

[40] Suchan B (2013). Reduced connectivity between the left fusiform body area and the extrastriate body area in anorexia nervosa is associated with body image distortion. Behavioural Brain Research.

[41] Vocks S (2010). Effects of body image therapy on the activation of the extrastriate body area in anorexia nervosa: an fMRI study. Psychiatry Research: Neuroimaging.

[42] Harel A (2016). What is special about expertise? Visual expertise reveals the interactive nature of real-world object recognition. Neuropsychologia.

[43] McGugin RW, Newton AT, Gore JC, Gauthier I (2014). Robust expertise effects in right FFA. Neuropsychologia.

[44] Rossion B, Gauthier I, Goffaux V, Tarr MJ, Crommelinck M (2002). Expertise training with novel objects leads to left-lateralized facelike electrophysiological responses. Psychological Science.

[45] Scott LS, Tanaka JW, Sheinberg DL, Curran T (2006). A reevaluation of the electrophysiological correlates of expert object processing. Journal of cognitive neuroscience.

[46] Scott LS, Tanaka JW, Sheinberg DL, Curran T (2008). The role of category learning in the acquisition and retention of perceptual expertise: A behavioral and neurophysiological study. Brain research.

[47] Calvo-Merino B, Grèzes J, Glaser DE, Passingham RE, Haggard P (2006). Seeing or doing? Influence of visual and motor familiarity in action observation. Current biology.

[48] Anllo‐Vento L, Luck SJ, Hillyard SA (1998). Spatio‐temporal dynamics of attention to color: Evidence from human electrophysiology. Human brain mapping.

[49] Zani, A. & Proverbio, A. M. Cognitive electrophysiology of mind and brain. In *The cognitive electrophysiology of mind and brain*, pp. 3–12 (Academic Press, 2003).

[50] Li K, Liu YJ, Qu F, Fu X (2016). Neural activity associated with attention orienting triggered by implied action cues. Brain research.

[51] Stahl J, Wiese H, Schweinberger SR (2008). Expertise and own-race bias in face processing: an event-related potential study. Neuroreport.

[52] Picton TW (1992). The P300 wave of the human event-related potential. Journal of clinical neurophysiology.

[53] Polich J (2007). Updating P300: an integrative theory of P3a and P3b. Clinical neurophysiology.

[54] Tanaka JW, Curran T, Porterfield AL, Collins D (2006). Activation of preexisting and acquired face representations: the N250 event-related potential as an index of face familiarity. Journal of Cognitive Neuroscience.

[55] Thorpe S, Fize D, Marlot C (1996). Speed of processing in the human visual system. Nature.

[56] Yang J, Guan L, Dedovic K, Qi M, Zhang Q (2012). The neural correlates of implicit self-relevant processing in low self-esteem: An ERP study. Brain research.

[57] Jin H (2011). Event-related potential effects of superior action anticipation in professional badminton players. Neuroscience letters.

[58] Aleong R, Paus T (2010). Neural correlates of human body perception. Journal of Cognitive Neuroscience.

[59] Downing PE, Peelen MV (2011). The role of occipitotemporal body-selective regions in person perception. Cognitive Neuroscience.

[60] Downing PE, Peelen MV (2016). Body selectivity in occipitotemporal cortex: Causal evidence. Neuropsychologia.

[61] Minnebusch DA, Daum I (2009). Neuropsychological mechanisms of visual face and body perception. Neuroscience & Biobehavioral Reviews.

[62] Bocquillon P (2014). The spatiotemporal dynamics of early attention processes: a high-resolution electroencephalographic study of N2 subcomponent sources. Neuroscience.

[63] Folstein JR, Van Petten C (2008). Influence of cognitive control and mismatch on the N2 component of the ERP: a review. Psychophysiology.

[64] Hopf JM (2006). The neural site of attention matches the spatial scale of perception. Journal of Neuroscience.

[65] Wang H, Ip C, Fu S, Sun P (2017). Different underlying mechanisms for face emotion and gender processing during feature-selective attention: Evidence from event-related potential studies. Neuropsychologia.

[66] Wong ACN, Palmeri TJ, Rogers BP, Gore JC, Gauthier I (2009). Beyond shape: how you learn about objects affects how they are represented in visual cortex. PloS one.

[67] Folstein JR, Monfared SS, Maravel T (2017). The effect of category learning on visual attention and visual representation. Psychophysiology.

[68] Amoruso L, Couto JB, Ibanez A (2011). Beyond Extrastriate Body Area (EBA) and Fusiform Body Area (FBA): context integration in the meaning of actions. Frontiers in Human Neuroscience.

[69] Bonini, L. The extended mirror neuron network anatomy, origin, and functions. *Neuroscientist* 1–12 (2016). 10.1177/1073858415626400 26747293

[70] Rizzolatti G, Sinigaglia C (2016). The mirror mechanism: a basic principle of brain function. Nature Reviews Neuroscience.

[71] Orlov T, Porat Y, Makin TR, Zohary E (2014). Hands in motion: an upper-limb-selective area in the occipitotemporal cortex shows sensitivity to viewed hand kinematics. Journal of Neuroscience.

[72] Kühn S, Keizer AW, Rombouts SA, Hommel B (2011). The functional and neural mechanism of action preparation: roles of EBA and FFA in voluntary action control. Journal of Cognitive Neuroscience.

[73] Valyear KF, Culham JC (2010). Observing learned object-specific functional grasps preferentially activates the ventral stream. Journal of Cognitive Neuroscience.

[74] Lingnau A, Petris S (2012). Action understanding within and outside the motor system: the role of task difficulty. Cerebral Cortex.

[75] Lingnau A, Downing PE (2015). The lateral occipitotemporal cortex in action. Trends in cognitive sciences.

[76] Cross ES, Mackie EC, Wolford G, Hamilton AFDC (2010). Contorted and ordinary body postures in the human brain. Experimental brain research.

[77] Cross ES (2009). Sensitivity of the action observation network to physical and observational learning. Cereb. Cortex.

[78] Calvo-Merino B, Glaser DE, Grèzes J, Passingham RE, Haggard P (2005). Action observation and acquired motor skills: an fMRI study with expert dancers. Cereb. Cortex.

[79] Urgesi C, Calvo-Merino B, Haggard P, Aglioti SM (2007). Transcranial magnetic stimulation reveals two cortical pathways for visual body processing. Journal of Neuroscience.

[80] Calvo-Merino B, Ehrenberg S, Leung D, Haggard P (2010). Experts see it all: configural effects in action observation. Psychol. Res. PRPF.

[81] Benoit RG, Gilbert SJ, Frith CD, Burgess PW (2011). Rostral prefrontal cortex and the focus of attention in prospective memory. Cerebral Cortex.

[82] Gilbert SJ (2006). Functional specialization within rostral prefrontal cortex (area 10): a meta-analysis. Journal of cognitive neuroscience.

[83] Ramnani N, Owen AM (2004). Anterior prefrontal cortex: insights into function from anatomy and neuroimaging. Nature Reviews Neuroscience.

[84] Halahalli HN, John JP, Lukose A, Jain S, Kutty BM (2015). Endogenous-cue prospective memory involving incremental updating of working memory: an fMRI study. Brain Structure and Function.

[85] Minamoto T, Yaoi K, Osaka M, Osaka N (2015). The rostral prefrontal cortex underlies individual differences in working memory capacity: An approach from the hierarchical model of the cognitive control. Cortex.

[86] Kietzmann TC, Ehinger BV, Porada D, Engel AK, König P (2016). Extensive training leads to temporal and spatial shifts of cortical activity underlying visual category selectivity. NeuroImage.

[87] Boutsen L, Humphreys GW, Praamstra P, Warbrick T (2006). Comparing neural correlates of configural processing in faces and objects: an ERP study of the Thatcher illusion. Neuroimage.

[88] Freunberger R, Klimesch W, Doppelmayr M, Höller Y (2007). Visual P2 component is related to theta phase-locking. Neuroscience letters.

[89] Lucas HD, Chiao JY, Paller KA (2011). Why some faces won’t be remembered: brain potentials illuminate successful versus unsuccessful encoding for same-race and other-race faces. Frontiers in Human Neuroscience.

[90] Neumann MF, End A, Luttmann S, Schweinberger SR, Wiese H (2015). The own-age bias in face memory is unrelated to differences in attention—Evidence from event-related potentials. Cognitive, Affective, & Behavioral Neuroscience.

[91] Vocks S (2010). Differential neuronal responses to the self and others in the extrastriate body area and the fusiform body area. Cognitive, Affective, & Behavioral Neuroscience.

[92] Amoruso L (2014). Time to tango: expertise and contextual anticipation during action observation. Neuroimage.

[93] Proverbio AM, Orlandi A, Pisanu F (2016). Brain processing of consonance/dissonance in musicians and controls: a hemispheric asymmetry revisited. European Journal of Neuroscience.

[94] Proverbio AM, Cozzi M, Orlandi A, Carminati M (2017). Error-related negativity in the skilled brain of pianists reveals motor simulation. Neuroscience.

[95] Oostenveld R, Praamstra P (2001). The five percent electrode system for high-resolution EEG and ERP measurements. Clinical neurophysiology.

[96] Picton TW (2000). Guidelines for using human event-related potentials to study cognition: recording standards and publication criteria. Psychophysiology.

[97] Palmero-Soler E, Dolan K, Hadamschek V, Tass PA (2007). SwLORETA: a novel approach to robust source localization and synchronization tomography. Physics in medicine and biology.

